# Interannual differences in common eider duck exposure to avian influenza viruses at an Arctic colony

**DOI:** 10.1093/conphys/coag033

**Published:** 2026-05-09

**Authors:** Jennifer F Provencher, Andre Morrill, Holly L Hennin, Jolene A Giacinti, Yohannes Berhane, Dmytro Zhmendak, Wanhong Xu, Oliver P Love, Mark R Forbes, H Grant Gilchrist

**Affiliations:** Science and Technology Branch, Environment and Climate Change Canada, 1125 Colonel By Drive, Raven Road, Ottawa, ON, K1S 5B6, Canada; Science and Technology Branch, Environment and Climate Change Canada, 1125 Colonel By Drive, Raven Road, Ottawa, ON, K1S 5B6, Canada; Science and Technology Branch, Environment and Climate Change Canada, 1125 Colonel By Drive, Raven Road, Ottawa, ON, K1S 5B6, Canada; Department of Biology, University of Windsor, 401 Sunset Avenue, Windsor, ON, N9B 3P4, Canada; Science and Technology Branch, Environment and Climate Change Canada, 867 Lakeshore Road, Burlington, ON, L7S 1A1, Canada; National Centre for Foreign Animal Disease, Canadian Food Inspection Agency, 1015 Arlington Street, Winnipeg, MB, R3E 3M4, Canada; National Centre for Foreign Animal Disease, Canadian Food Inspection Agency, 1015 Arlington Street, Winnipeg, MB, R3E 3M4, Canada; National Centre for Foreign Animal Disease, Canadian Food Inspection Agency, 1015 Arlington Street, Winnipeg, MB, R3E 3M4, Canada; Department of Biology, University of Windsor, 401 Sunset Avenue, Windsor, ON, N9B 3P4, Canada; Department of Biology, Carleton University, 1125 Colonel By Drive, Ravan Road, Ottawa, ON, K1S 5B6, Canada; Science and Technology Branch, Environment and Climate Change Canada, 1125 Colonel By Drive, Raven Road, Ottawa, ON, K1S 5B6, Canada

**Keywords:** Avian influenza virus, disease ecology, longitudinal monitoring, seroprevalence

## Abstract

Avian Influenza viruses (AIVs) regularly circulate among migratory birds, but starting in late 2021 an incursion of highly pathogenic AIV (HPAIV; H5N1 clade 2.3.4.4b) was detected widely in North American wild birds. Understanding exposure of migratory birds to AIV can be assessed through detection of anti-AIV antibodies, which indicate prior infection and survival. We used archived plasma samples from a long-term population monitoring programme to examine trends in prior AIV infection (as indicated by antibody detection) in female common eider duck (*Somateria mollissima borealis*) across years pre- and post-HPAIV detection in Canada (2018, 2019, 2022, 2023 and 2024). Anti-AIV antibodies have been assessed at this colony previously; therefore, we also combined the results from our analyses with previous data to extend the temporal trend analyses back to 2007. While we found that anti-nucleoprotein (NP) antibody positivity was relatively high across all five years, with observed proportions of seropositive females ranging from 76.9% (in 2019) to 100% (2018, 2023 and 2024), we found that levels of detections of anti-NP antibodies have been generally higher since 2011, with lower levels observed in earlier years (e.g. 2007). Anti-H5 antibody positivity levels increased significantly between 2019 and both 2023 and 2024, reflecting the likely change in exposure with the occurrence of the 2021 HPAIV H5N1 incursion in North America. We found no differences in annual anti-H7 antibody positivity levels. Our results demonstrate that Arctic-breeding common eider ducks have experienced varying exposure to AIV over the study period, with increasing exposure to multiple types of AIV based on an increase in antibody detections in recent years.

## Introduction

Avian influenza viruses (AIVs) have been reported to circulate in wild migratory birds in North America since the 1950s/1960s (Lycett *et al*., 2019). The inter-agency surveillance of AIV in wild birds in Canada has tracked AIV over time with mostly detections of Low Pathogenicity AIV (LPAIV) since the programme started in 2005 with both passive (testing opportunistic samples) and active sampling (targeting groups or regions) approaches employed (Parmley *et al*., 2009). For example, previous detections of LPAIV have been reported in several groups of migratory birds between 2005 and 2021, mostly in waterfowl (Parmley *et al*., 2011; Soos *et al*., 2012; Humphreys *et al*., 2021; McLaughlin *et al*., 2024). Notably, in 2014/15 a High Pathogenicity AIV (HPAIV) incursion into North America resulted in outbreaks on poultry farms in two provinces, with a few detections in wild birds. More recently, in late 2021 an incursion of HPAIV (clade 2.3.4.4b) rapidly spread throughout migratory bird populations in North America, causing severe levels of morbidity and mortality in some wild species (Ramey *et al*., 2022; Giacinti *et al*., 2024b; Signore *et al*., 2025). The initial incursion of HPAIV in 2021 has been followed by reassortments and recurring incursions and dissemination of other strains of HPAIV, leading to widespread exposure of migratory birds to avian influenza viruses (AIVs) concurrently (Erdelyan *et al*., 2024; Rahman *et al*., 2024). Unlike previous AIVs in North America detected before 2021, the HPAIVs introduced in 2021 have resulted in widespread mass mortalities of species that have varying life history strategies in relation to breeding and foraging, and include colonial seabirds (e.g. northern gannets (*Morus bassanus*), common murres (*Uria aalge*), common eider (*Somateria mollissima*)) as well as waterfowl such as Canada geese (*Branta canadensis*) and snow geese (*Anser caerulescens*), which has led to concerns around population-level impacts for the species most vulnerable to the virus.

When birds are infected with AIV (and do not perish from it), it results in cell- and antibody-mediated adaptive immune responses. Serum (the fluid remaining when blood is clotted) and plasma (the liquid component of anticoagulated blood) can be used to detect antibodies in wild birds (Escandon *et al*., 2019; Giacinti *et al*., 2025) and have been shown to yield comparable results for antibody detection in some systems (Siev *et al*., 2011; Chentoufi *et al*., 2025). Antibody responses to AIV include those directed against the conserved nucleoprotein (NP), an internal viral antigen that is relatively stable across subtypes and is commonly used for broad serological detection of prior infection. Antibodies targeting the AIV hemagglutinin (HA) surface protein are subtype specific, reflecting variation across HA subtypes (e.g. H5). Previous AIV studies have shown that several species of wild ducks have shown high levels of anti-NP antibodies (Bevins *et al*., 2014; Hall *et al*., 2015).

Detection of antibodies in wild birds is influenced by host, pathogen and environmental factors, including infection history, timing since exposure and underlying host condition. Antibody kinetics can vary within species; for example, anti-HA antibodies (e.g. H5) antibodies may wane over shorter timeframes (weeks), whereas anti-NP antibodies can persist for longer periods (months) in ducks (Wight *et al*., 2024; Giacinti *et al*., 2024a). Given these factors that can influence anti-AIV antibodies levels, antibody results generally are expected to reflect recent exposure although the duration of detectability is likely to vary by species, age and antigenic subtype among other factors (Hill *et al*., 2016; Qi *et al*., 2021). Thus, antibodies can serve as a proxy for prior infection and provide a useful tool at the population scale to assess patterns of exposure (McLaughlin *et al*., 2025).

Waterfowl, including ducks and geese, have been identified as common hosts for AIVs (Fereidouni *et al*., 2010; Abao *et al*., 2013; van Dijk *et al*., 2014; Papp *et al*., 2017). Importantly, the North American Arctic represents an important overlap zone of the flyways where many migratory birds, including ducks and geese, converge on the northern breeding grounds, which can lead to viral mixing between the continents (Ramey *et al*., 2011; Reeves *et al*., 2013; Wilson *et al*., 2013). Common eiders are known to be asymptomatic carriers of LPAIV and to have antibodies for several months post exposure (Hall *et al*., 2015). Due to harvest of this species in North America, long-term monitoring has tracked population and health metrics over time, which has resulted in archiving samples for retrospective studies. This includes previous AIV surveillance at several of the main population monitoring sites in Canada.

To explore patterns in anti-AIV antibodies across years with and without widespread incursion events of Eurasian HPAIV in North America, we used archived plasma samples from Arctic-breeding common eider ducks (*Somateria mollissima borealis*; hereafter ‘eiders’) collected from one of Canada’s largest breeding colonies (Gilchrist *et al*., 2025). We expected to see an increase in anti-H5 antibodies in eiders in 2022, with a larger increase in 2023 due to the likely increase in H5N1 HPAIV exposure in the region, and as seen with the antibody levels in eiders eggs from the same region (McLaughlin *et al*., 2024). The viral detections of H7 AIVs have been low in wild birds in North America over this period, so we expected anti-H7 antibody detections in the eiders at Mittivik Island to be low between 2018 and 2024. For anti-NP antibodies, we extend the new data presented here using Hall *et al*. (2015) as the work was undertaken at the same location and using the same methods. Thus, based on previous work examining anti-NP antibodies in eggs (McLaughlin *et al*., 2024) and previous antibody work on this species, we expected to see a relatively high and constant level of anti-NP antibodies across the longer time period for which anti-NP data is available (2007–2024) as it is known that eiders are regularly exposed to AIVs (Hall *et al*., 2015).

## Materials and Methods

### Field collections and animal care considerations

Eiders were caught at the breeding colony on Mittivik Island (East Bay Migratory Bird Sanctuary) in Northern Hudson Bay (64°01′04″ N, 82°07′49″ W) as part of the long-term research programme (Gilchrist *et al*., 2025). Female eiders that breed at Mittivik Island spend the non-breeding season in waters off Greenland and Newfoundland each year (Mosbech *et al*., 2006; Steenweg *et al*., 2017). Each spring they return to northern Canada between May and June and lay their eggs between June and July (Gilchrist *et al*., 2025). The eiders returning to the area each year are caught using monofilament flight nets as they arrive on the island and before nest initiation. Upon capture, each female is banded to account for repeated sampling of the same individual. All appropriate animal care permits were in place and approved by both Environment and Climate Change Canada and the University of Windsor (Animal Use Permits 09-06, 11-06, 15-05, 19-11, 22-06) Animal Care Committees (see previous publications for details).

The original intent of collecting these samples was to examine a range of physiological metrics in relation to eider phenology and reproduction. Over the course of the programme at Mittivik Island, studies have looked at various physiological metrics including corticosterone (Harms *et al*., 2015), triglycerides (Hennin *et al*., 2015), and immunoglobulin Y (Provencher *et al*., 2016). Therefore, the additional testing of these samples for AIV-specific antibodies fell within the original permitting and consultation processes.

**Figure 1:**
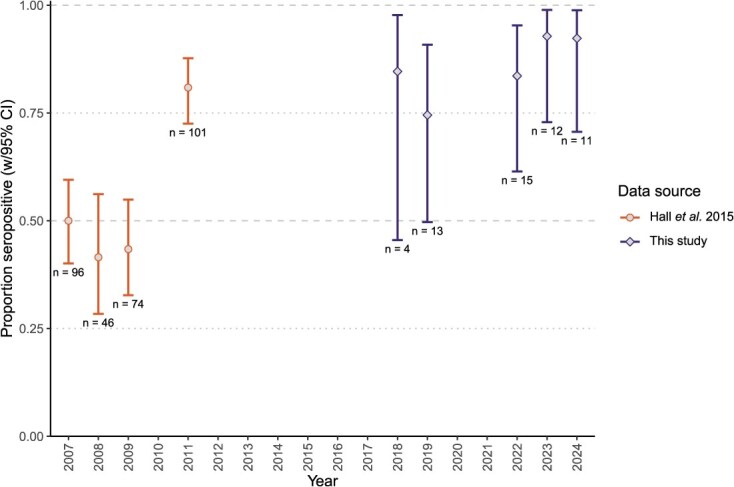
Estimated positivity levels of anti-NP antibodies in serum samples from female common eider ducks collected at Mittivik Island, Nunavut, across sampling years. Estimates are from a logistic regression with year as a categorical predictor. Error bars are 95% credible intervals calculated as quartile intervals. Estimates from 2011 and earlier are based on data from Hall *et al*. (2015)

When an adult female eider was caught, a small blood sample (maximum 1 ml) was taken immediately from the tarsal vein after capture at the net and within 3 min of the birds hitting the net using a 23G thin-wall, 1-in. (c. 25-mm) needle attached to a heparinized 1-ml syringe (Fig. 1; Hennin *et al*., 2015). All blood samples were kept at 4°C and centrifuged at 10 000 rpm for 10 min within 6 h of collection. Upon separation, the plasma component of the blood was collected and stored at −20°C for further analysis.

Subsequent to annual sampling and various projects, any remaining plasma was archived at the University of Windsor (Integrative Avian Ecology Lab) at −80°C with minimal freeze–thaw cycles. Given the HPAIV incursions into North America starting in late 2021, we accessed stored samples from the project archive for Mittivik Island to explore patterns in AIV exposure over time. We were limited in our selection of years and individuals as we could only access samples that were greater than 150 μl in volume which is the quantity needed for the assays as described below. Based on these criteria, we selected a total of 55 samples from 2018 (*n* = 4), 2019 (*n* = 13), 2022 (*n* = 15), 2023 (*n* = 12), and 2024 (*n* = 11) from the archive. In each year, all females were caught between 19 June and 30 June. No samples were available from 2020 and 2021 due to field programmes not being implemented due to travel restrictions during the COVID-19 pandemic.

### Laboratory methods

Samples were shipped frozen at −70 to −90°C to ECCC’s National Wildlife Research Centre (NWRC) in Ottawa for antibody testing. Plasma samples were initially processed at the NWRC where AIV anti-NP antibodies were detected using the IDEXX AI MultiS Screen Ab test (IDEXX Canada, Product # 99-12119) as per manufacturer’s instructions (Brown, 2010). A sample to negative control (or signal to noise) ratio (S/N) <0.5 was considered positive for anti-NP antibodies.

For this project, all 55 samples were sent to the Canadian Food Inspection Agency’s National Centre for Foreign Animal Disease (NCFAD) to screen for presence of hemagglutinin-specific anti-AIV antibodies (Hochman *et al*., 2023). Anti-H5 and anti-H7 antibodies at NCFAD were detected using previously described methods (Yang *et al*., 2010; Hochman *et al*., 2023).

### Statistical approaches

To explore patterns in antibodies over time, positivity for anti-NP, anti-H5 and anti-H7 antibodies was modelled separately using logistic regression (binomial family, logit link), with year included as the sole predictor and treated as a categorical factor. Previous work at the Mittivik Island site also tested for anti-Np antibodies using the same IDEXX kits; therefore, we examined patterns of anti-Np antibodies using our own data, as well as previous data from this site (2007–2011). Specifically, from the Hall *et al*. (2015) paper we used the Summer-Nunavut data as this corresponds to Mittivik Island (while the spring samples correspond to Cape Dorset/Kinggait, Nunavut).

All antibody data (both Hall *et al*. (2015) data and the new data presented here) were initially modelled using maximum-likelihood frequentist methods. Since maximum-likelihood binomial models showed near-complete separation for NP positivity (some years had 100% seropositivity) which produced implausibly large logit estimates and standard errors, we fit the logistic regressions for anti-NP antibodies in a Bayesian framework using weakly informative normal (mean = 0, SD = 1.5) priors to regularize estimates and contrasts.

Temporal models were compared to null models using either Akaike’s Information Criterion corrected for small samples (maximum likelihood–fitted models; Burnham and Anderson, 2002) or expected log pointwise predictive density estimated by leave-one-out cross-validation (ELPD-LOO, Bayesian models; Vehtari *et al*., 2017). ELPD-LOO was computed using Pareto-smoothed importance sampling (Vehtari *et al*., 2024). Where ELPD-LOO supported interannual variation in seropositivity, pairwise differences between yearly effects were estimated from the posterior distributions of the indexed year parameters. All analyses were performed in R (version 4.3.2; RCoreTeam, 2023); Bayesian models were coded in Stan (Stan Development Team, 2024) and run using four chains (1500 warm-up iterations and 1000 sampling iterations per chain; 4000 posterior samples total) via the ‘cmdstanr’ package (Gabry and Češnovar, 2022). Pairwise comparisons for frequentist models were calculated using the ‘marginaleffects’ package with Holm corrections for multiple comparisons (Arel-Bundock, 2024). *R^* values of all parameters in Bayesian models were <1.01, indicating good mixing of MCMC chains. All parameter posterior effective sample sizes were >500 per chain.

## Results

**Figure 2:**
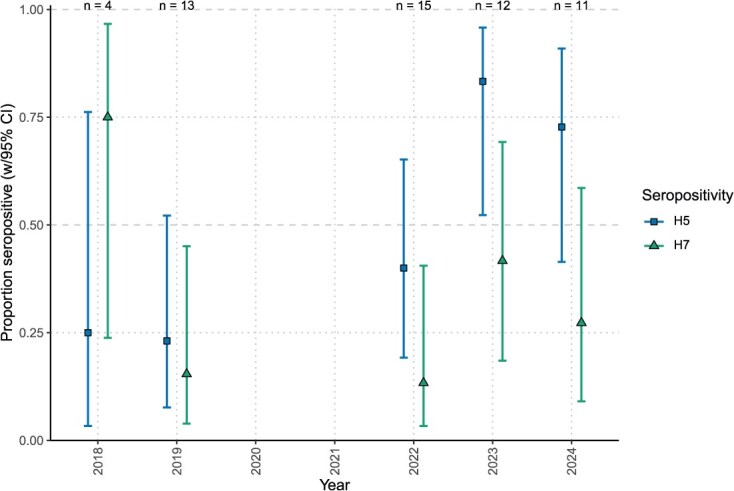
Estimated positivity levels of anti-H5 and anti-H7 antibodies in serum samples from female common eider ducks collected at Mittivik Island, Nunavut, before and after the HPAIV incursion in North America in late 2021. Estimates for each antibody type are from logistic regressions with year as a categorical predictor. Note that the model of anti-H7 positivity as dependent on year did not outperform a null model of constant positivity over time. Error bars are 95% confidence intervals

Anti-NP antibody positivity in female eiders was relatively high across all five years analysed, with observed proportions of seropositive females ranging from 76.9% (in 2019; 10/13) to 100% (2018, 2023 and 2024) (Table 1, Fig. 1). Observed annual anti-H5 and anti-H7 antibody positivity levels appeared more variable (Table 1); however, only models of anti-H5 positivity predicted by sampling year performed better than the null model, and therefore tests of interannual variation in anti-H7 positivity were not pursued further (Table 2, Fig. 2). After correcting for multiple comparisons, *post hoc* pairwise contrasts of the annual anti-H5 positivity estimates indicated significant differences between 2019 and both 2023 (*z* = 3.79, Holm-corrected *P* = 0.001) and 2024 (*z* = 2.79, Holm-corrected *P* = 0.048); other comparisons of annual estimates were nonsignificant.

**Table 1:** Estimated seropositivity for anti-NP, H5 and H7 antibodies measured in the plasma of breeding female common eiders (*Somateria mollissima borealis*) at Mittivik Island, Nunavut across years of sampling

Year	Positivity (95% CI; *n*_positive_/*n*_total_)		
	Anti-NP	Anti-H5	Anti-H7
2018	0.846 (0.455–0.977; 4/4)	0.250 (0.034–0.762; 1/4)	0.750 (0.238–0.966; 3/4)
2019	0.745 (0.497–0.908; 10/13)	0.231 (0.076–0.522; 3/13)	0.154 (0.039–0.451; 2/13)
2022	0.836 (0.614–0.953; 13/15)	0.400 (0.192–0.652; 6/15)	0.133 (0.034–0.405; 2/15)
2023	0.928 (0.729–0.989; 12/12)	0.833 (0.523–0.958; 10/12)	0.417 (0.185–0.692; 5/12)
2024	0.923 (0.706–0.988; 11/11)	0.727 (0.414–0.910; 8/11)	0.273 (0.090–0.586; 3/11)

Errors on estimates from best-fitting models are 95% credible intervals for NP and 95% confidence intervals for H5 and H7. Observed positivity (number of samples positive/total number of samples) is also provided.

**Table 2:** Comparisons of common eider (*Somateria mollissima borealis*) anti-H5, -H7 and -NP antibody seropositivity logistic regressions, formulated with positivity dependent on sampling year as a categorical predictor or as a null model

Antibody	Model	AICc	ΔAICc	ELPD (SE)	ΔELPD (SE)	*n*_param._ (SE)
Anti-H5	**Year model**	**73.66**				**5**
	Null model	78.30	4.64			1
Anti-H7	**Null model**	**66.53**				**1**
	Year model	67.86	1.33			5
Anti-NP	**Year model**			**−219.45 (7.36)**		**5.95 (0.55)**
	Null model			−247.86 (4.64)	−28.41 (6.74)	1.01 (0.03)

Anti-H5 and -H7 models were compared using Akaike’s Information Criterion corrected for small sample sizes (AICc; lower values preferred) and Bayesian anti-NP antibody models were compared using expected log pointwise predictive densities (ELPD; higher values preferred). Best-fitting models are indicated in bold. Values in parentheses are standard errors (SEs). *n*_param_ indicates the number of model parameters; for Bayesian models, this is the estimated effective number of parameters, provided with the standard error.

When incorporating anti-NP antibody positivity data from Hall *et al*. (2015), we found strong evidence for interannual variation in positivity estimates (Table 2). Contrasts of annual estimates indicated higher anti-NP antibody positivity in 2022 through 2024 when compared to 2007 through 2009 (contrast 95% CI not overlapping zero; Fig. S1). There was additional strong evidence that anti-NP antibody positivity levels in 2018 and 2019 were higher than those of 2008 and 2009, and moderate-to-strong evidence (contrast 90% CI not overlapping zero) that 2018–19 levels were higher than those in 2007 (Fig. S1). However, there was no evidence that levels from 2018 to 2024 were higher than the positivity in 2011, and 2011 anti-NP antibody positivity was higher than levels in 2007, 2008 and 2009 (Fig. 1, Fig. S1).

## Discussion

We found that anti-Np antibodies were commonly detected in female eiders at Mittivik Island in northern Hudson Bay (observed percentages from 76.9% to 100%) throughout the period of this study (2018 to 2024). Previous reports have documented anti-NP antibodies between 39% and 81% between 2007 and 2011 at the same colony (Hall *et al*., 2015). While sample sizes differ greatly between the sampling years, our results support previous work that Arctic-breeding eiders are exposed to AIV.

When we examined the data from Hall *et al*. (2015) and our current study, we found that the anti-NP antibodies in eiders at Mittivik Island were highest in more recent years. Contrary to our prediction, higher positivity levels were estimated from 2011 onwards, rather than exclusively in the most recent years (2023 and 2024). The samples from 2018 and 2019 were collected prior to the HPAIV incursions that started in North America in late 2021, and thus samples from 2022 to 2024 represent years when eiders throughout Canada had apparently experienced greater exposure to novel HPAIV. Instead, our results found that plasma samples in 2009 and earlier showed significantly lower detections of anti-NP antibodies compared to those from 2011 to 2024. This pattern was surprising given that we expected exposure to AIVs to increase in 2022 onwards in relation to the previously reported HPAIV outbreak in North America (McLaughlin *et al*., 2024; Giacinti *et al*., 2024b). Previous retrospective analyses using data from both common and thick-billed murres in Canada have shown higher levels of exposure to both AIV and H5 AIV from 2022 onwards (McLaughlin *et al*., 2024), but our reported rise in detection of anti-NP antibodies in Common eiders at Mittivik Island in the Canadian Arctic predates the change detected in murre exposure by a decade. Regardless of the timing, these studies suggest that AIV exposure has increased in multiple seabird species over the last several years.

It is important to note that the existing data from Hall *et al*. (2015) does not identify the sex of the eiders tested at Mittivik Island for anti-NP antibodies and likely includes samples from both male and female eiders as they are both caught as part of research at this site. We acknowledge that this difference in including one or both sexes of birds could impact our results, but this is unlikely for several reasons. First, the sample from 2011 (Hall *et al*., 2015; likely mixed sexes) shows similarly high levels of positivity to the 2018 to 2024 samples (this study; females only). If the level of positivity was greatly influenced by the sex ratio in the samples, we would have expected to see 2007 to 2011 grouping together (mixed sexes) and the 2018 to 2024 data grouping together (females only) with a significant difference between the two groupings. Second, male and female eiders typically establish pair bonding on the over-wintering grounds, which precedes the summer sampling by several months (Steenweg *et al*., 2017). Therefore, when the males and females arrive on the island, both sexes have experienced similar environmental conditions and opportunities for viral exposure in the months prior to arrival at the colony, which may be reflected in comparable antibody profiles. This pattern contrasts several other marine bird species, including murres, that may have males and females arriving at the colonies at different time periods and thus reflecting a difference in pre-breeding conditions (Dittmann and Becker, 2003; Ismar-Rebitz *et al*., 2020). It is also possible that the year 2011 was dominated by females in the sample collection, which is why there are similar results to those years in which we only analysed female samples. In our experiences at this site, it is unlikely, but future work should consider how antibodies may differ with sex in birds with similar and dissimilar migratory strategies (and thus opportunities for AIV exposure).

When we examined the anti-H5 antibodies from 2018 to 2024, we found that the samples collected in 2023 and 2024, when there was a widespread H5N1 HPAIV incursion, showed significantly higher detection levels than in 2019, i.e. higher than the only pre-2022 year associated with a sample size greater than *n* = 4. This pattern matched our predictions given that a known increase in AIV H5N1 virus started in late 2021, with many populations of migratory birds exposed to the virus during the spring of 2022. Prior to late 2021, LPAIV H5 detections were uncommon in North America, thus this increase in anti-H5 antibody detections suggests that they can be attributed to the 2021 incursion. It is also important to highlight that there have been multiple incursions of Eurasian H5 HPAIV to North America bird populations since 2021. Genetic analyses have revealed that clade 2.3.4.4b H5N1 dominated detections in wild birds in 2021 and 2022 (Signore *et al*., 2025). However, since that time, additional clade 2.3.4.4b viruses, including H5N5 and H5N1, have been detected across Canada (Erdelyan *et al*., 2024; Rahman *et al*., 2024). Thus, while we detected anti-H5 antibodies in eiders, the available data do not allow us to determine which H5 virus these antibodies were generated against. Regardless, these antibody results confirm that eiders breeding in northern Hudson Bay during this period have experienced greater exposure to H5Nx viruses in more recent years, at least based on five years of monitoring data.

In addition to the plasma samples examined within this study, a total of 86 eiders were swabbed for AIV at Mittivik Island in 2022 to assess active viral infection; a subset of these 86 were birds sampled for AIV antibodies and reported in this study. This sampling occurred in the first year following the incursion of clade 2.3.4.4b viruses into North America, when H5 HPAIV detections in wild birds were high in North America (Giacinti *et al*., 2024b). While no AIV matrix gene detections were observed in samples tested from Mittivik Island in 2022, our results suggest that the eiders breeding on the island were exposed to the virus prior to breeding. Unfortunately, viral sampling in previous and subsequent years has been limited due to the capacity of the field crews, so it is difficult to compare the antibody data to viral detections in other years as previously done in Hall *et al*. (2015). What we know from other regions, including Québec and the maritime regions in eastern Canada, is that eiders (*dresseri* subspecies) were widely exposed to the virus in the first year of the outbreak (2022), with high levels of mortality detected at some colonies (Avery-Gomm *et al*., 2024; Giacinti *et al*., 2024b). Eiders have also shown widespread exposure to AIV both in eastern and northern Canada assessed via antibodies in eggs in 2022 to 2023 (McLaughlin *et al*., 2025). Interestingly, as one of the focal species with egg collections across almost 30 degrees of latitude in 2023, eider eggs showed a negative correlation between anti-H5 antibodies and latitude (more southern colonies had higher levels), but no relationship between anti-NP antibodies and latitude (McLaughlin *et al*., 2025). Cumulatively, these findings suggest that eiders frequently show evidence of prior infection with AIV. Because the virus has not been detected during the breeding season at this colony through surveillance efforts, antibody screening provides an important source of information on past infection and potential implications for survival.

It is not clear why common eiders are generally detected to be more frequently exposed to AIVs as compared to other sympatric species. Ecologically, common eiders are colonial nesting birds that spend the vast majority of their lives in the marine environment and return to land only during the breeding season (i.e. the definition of seabirds; Gaston, 2004). But previous research demonstrates that eiders show much higher levels of AIV infection compared with other seabirds that breed in the same areas (Giacinti *et al*., 2024b; McLaughlin *et al*., 2025). Taxonomically, eiders are waterfowl, a group that is widely known to be asymptomatic carriers of AIV (Wilson *et al*., 2013), which may correlate to physiological traits that respond to AIV exposure and allow for more asymptomatic carriers in the population. Previous work examining how traits contributed to patterns of AIV mortality suggested that both breeding phenology, inter-nest distances and interactions at sea with conspecifics all correlated in species with similar AIV mortality (McPhail *et al*., 2025). Future work should consider how both ecological and physiological traits may contribute to how species are exposed to AIV and how sympatric species respond over time to repeated exposure to a pathogen.

Beyond individual-level patterns in antibody responses, common eiders are susceptible to several pathogens capable of causing severe population-level impacts. For example, previous work has documented the outbreaks of avian cholera (caused by the bacteria *Pasteurella multocida*) in eastern and northern Canada. While avian cholera had been documented in southern Canada and the USA dating back to the 1940s, it was detected in Arctic-breeding common eider colonies in the early 2000s for the first time by Inuit hunters (Henri *et al*., 2018). This generated new research work at Mittivik Island which tracked the disease impacts on reproduction and population metrics with early modelling indicating that avian cholera would lead to a colony collapse if the annual outbreaks at the colony persisted at the same levels as observed in the first four years (2005 to 2008) (Descamps *et al*., 2012). However, continued monitoring of this colony showed that rather than the colony collapsing due to continued avian cholera outbreaks generating both adult mortality and years of reproductive failure, instead herd immunity likely led to an epidemic fadeout by 2012 (van Dijk *et al*., 2021). While LPAIV has been detected in the colony previously (Hall *et al*., 2015), and considering that this study indicates increased exposure to AIVs over time, no unusual mortality events related to AIV have been observed at Mittivik Island despite annual monitoring up to 2025, suggesting that AIV is likely to have a detectable population-level impact at this time.

This study demonstrates the benefit of utilizing both archived physiological samples and previously published data to examine how migratory birds are exposed to viruses over decadal time scales. Our results further demonstrate that Arctic-breeding common eiders are regularly exposed to AIVs, and that exposure changes over time and in relation to how AIV is circulating in migratory bird populations. Our findings also demonstrate that most eiders breeding at East Bay Island have evidence of prior AIV infection. These results indicate that eiders breeding in the Hudson Bay–Hudson Strait region generally initiate breeding with anti-AIV antibodies already present, likely reflecting previous exposure to multiple AIV subtypes.

## Supplementary Material

Web_Material_coag033

## Data Availability

All antibody data generated for this study are available in the Supplemental material.
