# Supplementary material for: Interannual differences in common eider duck exposure to avian influenza viruses at an Arctic colony
Source: Conserv Physiol. 2026 May 9;14(1):coag033. doi: 10.1093/conphys/coag033 (PMC13159724; doi:10.1093/conphys/coag033)
Supplement: Web_Material_coag033 [file web_material_coag033.zip › EBI_COEI_AIV_serum_ms_revision_April_2026_SupMat_coag033.pdf]

Supplemental Material

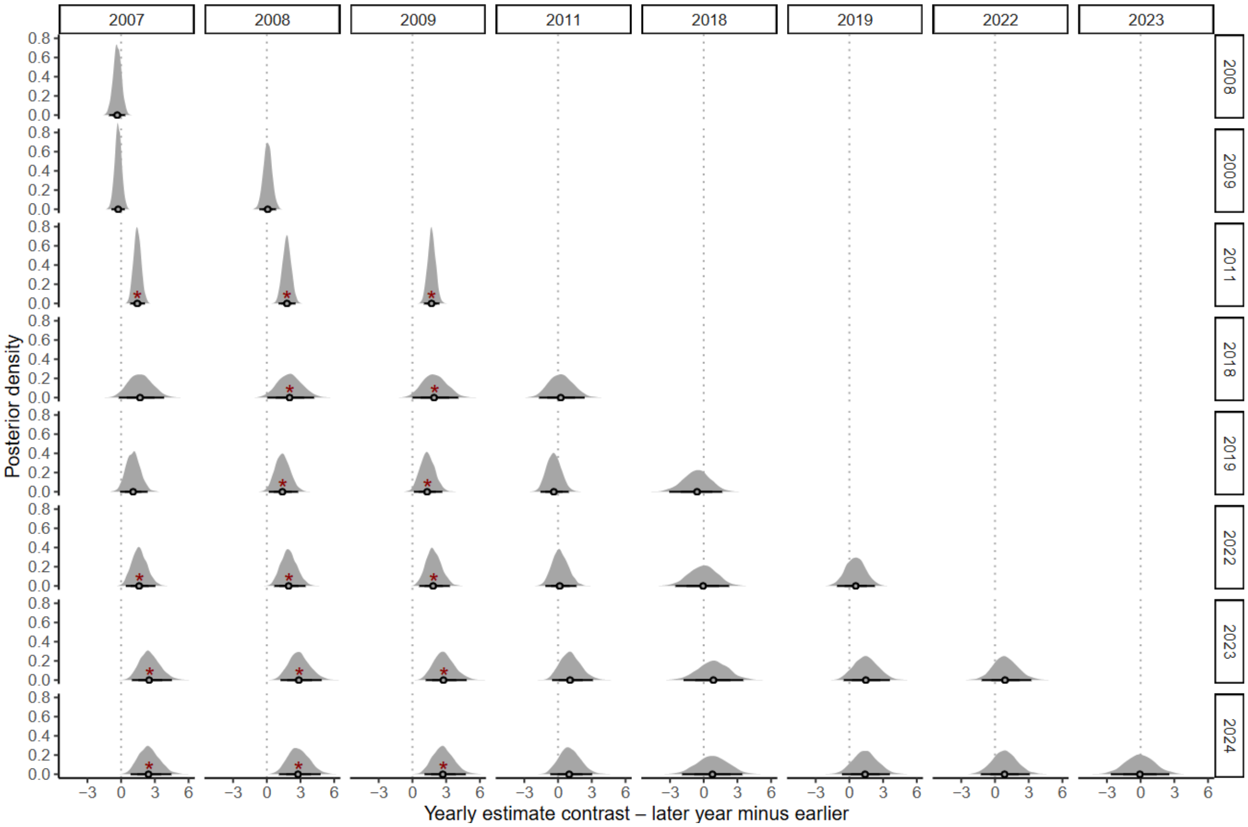

Figure S1. Pairwise contrasts of posterior estimates for the year effect on female Common eider anti-NP antibody seropositivity (later year minus earlier year estimate on the logit scale). Points and error bars below density estimates represent mean contrasts and 95% credible intervals (CIs) calculated as quantile intervals. Red asterisks indicate comparisons where the 95% CI did not include zero.
